# A novel mushroom (*Auricularia polytricha*) glycoprotein protects against lead-induced hepatoxicity, promotes lead adsorption, inhibits organ accumulation of lead, upregulates detoxifying proteins, and enhances immunoregulation in rats

**DOI:** 10.3389/fnut.2023.1144346

**Published:** 2023-04-06

**Authors:** Shuang Zhao, Yi Gao, Hexiang Wang, Yangyang Fan, Pan Wang, Wenting Zhao, Jack Ho Wong, Dan Wang, Xiaoyan Zhao, Tzi Bun Ng

**Affiliations:** ^1^Institute of Agri-Food Processing and Nutrition, Institute of Plant Protection, Beijing Academy of Agriculture and Forestry Sciences, Beijing Key Laboratory of Fruits and Vegetable Storage and Processing, Beijing, China; ^2^Department of Stomatology, Beijing Xicheng District Health Care Center for Mothers and Children, Beijing, China; ^3^State Key Laboratory for Agrobiotechnology and Department of Microbiology, China Agricultural University, Beijing, China; ^4^School of Health Sciences, Caritas Institute of Higher Education, Hong Kong, China; ^5^School of Life Sciences, Faculty of Science, The Chinese University of Hong Kong, Hong Kong, China

**Keywords:** *Auricularia polytricha*, detoxification, glycoprotein, lead elimination activity, proteomics

## Abstract

**Introduction:**

Lead is a ubiquitous environmental and industrial pollutant. Its nonbiodegradable toxicity induces a plethora of human diseases. A novel bioactive glycoprotein containing 1.15% carbohydrate, with the ability of adsorbing lead and effecting detoxification, has been purified from Auricularia polytricha and designated as APL. Besides, its mechanisms related to regulation of hepatic metabolic derangements at the proteome level were analyzed in this study.

**Methods:**

Chromatographic techniques were utilized to purify APL in the current study. For investigating the protective effects of APL, Sprague-Dawley rats were given daily intraperitoneal injections of lead acetate for establishment of an animal model, and different dosages of APL were gastrically irrigated for study of protection from lead detoxification. Liver samples were prepared for proteomic analyses to explore the detoxification mechanisms.

**Results and discussion:**

The detoxifying glycoprotein APL displayed unique molecular properties with molecular weight of 252-kDa, was isolated from fruiting bodies of the edible fungus A. polytricha. The serum concentrations of lead and the liver function biomarkers aspartate and alanine aminotransferases were significantly (p<0.05) improved after APL treatment, as well as following treatment with the positive control EDTA (300 mg/kg body weight). Likewise, results on lead residue showed that the clearance ratios of the liver and kidneys were respectively 44.5% and 18.1% at the dosage of APL 160 mg/kg, which was even better than the corresponding data for EDTA. Proteomics disclosed that 351 proteins were differentially expressed following lead exposure and the expression levels of 41 proteins enriched in pathways mainly involved in cell detoxification and immune regulation were normalized after treatment with APL-H. The results signify that APL ameliorates lead-induced hepatic injury by positive regulation of immune processing, and suggest that APL can be applied as a therapeutic intervention of lead poisoning in clinical practice. This report represents the first demonstration of the protective action of a novel mushroom protein on lead-elicited hepatic toxicity.

## 1. Introduction

Heavy metals are commonly applied, in line with their specific physical features, in industrial production such as alloy production, battery manufacture, building industry, ceramics, corrosion and acid-resistant materials, cosmetics, hair dyes, paints, water pipes, and weaponry (1–4). Heavy metals accumulate in air, water, soil and plants for a long duration, enter the human body through inhalation and the diet, leading to intoxication in humans. Consequently, persistent heavy metal contamination has become a worldwide threat to human existence and the environment (5).

Lead (Pb) is one of the principal heavy metal contaminants. Lead exposure contributes to metabolic disorders in various tissues encompassing the liver, kidneys, gonads, blood and the central nervous system of mammals, which are associated with respiratory, neurologic, hematological, gastrointestinal, cardiovascular, genitourinary, circulatory and immunological pathologies in mammals including humans (6–8). Lead is regarded as a highly poisonous environmental pollutant. Lead which is consumed undergoes hepatic conjugation before passage to the kidneys, in which a portion is removed. Thus, the liver is the organ affected to the greatest extent from ingestion and/or inhalation exposure (1). Previous studies reported that long-term lead exposure inflicted oxidative damage in the liver, kidneys and other organs as evidenced by generation of reactive oxygen species and different free radicals, inhibition of activities of antioxidant enzymes and enhancement of lipid peroxidation (9–12). However, efficacious therapy of lead exposure with minimal side effects and metabolic regulation still necessitates more research and studies. The molecular actions of protective extracts toward the liver are unclear, and the mechanisms related to regulation of hepatic metabolic derangements brought about by bioactive compounds at the proteome level have not been fully elucidated.

The use of herbal and mushroom extracts containing polysaccharides, proteins, polyphenols and flavonoids for preventing and facilitating recovery from diseases induced by environmental toxicants has captured considerable attention (13–16). *A. polytricha* is a medicinal and culinary fungus extensively cultivated worldwide, and its fruiting bodies and extracts have frequently been deployed in traditional medicine or included in the diet in Asian countries because of therapeutic value (17). Previous studies reported that *A. polytricha* polysaccharide and protein extracts exhibit a myriad of beneficial effects, encompassing antioxidant, anti-hypolipidemic, hepatoprotective and renoprotective bioactivities, especially biosorption of heavy metal ions from the external environment (18–26), such as copper, zinc, lead and mercury ions. In our previous study, the fruiting bodies and crude extract of *A. polytricha* exhibited potent lead ion adsorption capabilities, which was consistent with other research reports (22–26). Thus, we hypothesize that the *A. polytricha* extract would adsorb the lead residues in circulation and eliminate them from the body. The hepatoprotective activity of *A. polytricha in vivo* suggests that it may protect the liver and the kidneys from damage elicited by chronic exposure to lead.

*Auricularia polytricha* extract is known to exert a biosorption effect toward lead from a contaminated environment. However, there is a lack of information on the therapeutic effect of application of a purified *A. polytricha* component on lead-induced toxicity in the blood circulation and in the liver. In view of this, the present investigation was undertaken to assess the lead eliminating activity and hepatorenal-protective role of the isolated *A. polytricha* glycoprotein in rats chronically exposed to lead, and to analyze the molecular responses, expression of enzymes and possible signaling pathway associated with regulation of liver metabolism by deploying proteome technique, in order to expand the application of the therapeutic potentials of *A. polytricha* in pharmaceutical and functional food industries.

The present article represents the first report that administration of a glycoprotein derived from a medicinal-culinary mushroom was capable of attenuating the toxicity of lead as attested to by the eternal appearance, feed consumption and body weight of the experimental animals, blood lead concentration, serum activities of enzymatic markers of hepatic function, and hepatic and renal concentrations of lead. The study also disclosed that an immune mechanism was implicated in the protective effect of the mushroom glycoprotein against lead toxicity. Hence the mushroom glycoprotein manifests therapeutic potential in managing lead intoxication.

## 2. Materials and methods

### 2.1. Materials and reagents

Fresh fruiting bodies of *A. polytricha* were collected in the suburb of Beijing, China, and were sun-dried and crushed to produce a fine powder. The assay kits for serum indexes were obtained from Nanjing Jiancheng Bioengineering Institute and Applygen Technology Ltd. Standard monosaccharides were purchased from Merck (USA). DE-52 and Superdex-200 column were purchased from Solarbio (Beijing, China) and General Electric Company (GE, USA). All other chemicals and solvents applied were of analytical reagent grade.

### 2.2. Purification of APL

APL crude polysaccharide was extracted in accordance with the previously reported method (21). The crude APL was reconstituted in deionized water and loaded on a 1 cm × 30 cm DEAE- 52 column which had previously been equilibrated with deionized water. The column was eluted with 0 and then with 0.8 mol/L NaCl solution (flow rate: 1.5 mL/min). Based on the lead clearance capacity, the adsorbed peak enriched in polysaccharide was collected, concentrated, and further purified on an FPLC-Superdex 200 10/300 column which was eluted with 0.2 mol/L NH_4_HCO_3_ (pH 8.5) buffer using an AKTA Purifier (GE Healthcare), The polysaccharide content in the eluted fractions was quantitated by employing the phenol sulfuric acid method.

### 2.3. Determination of *in vitro* lead clearance capacity of APL

The polysaccharide sample obtained from the aforementioned purification steps was freeze-dried and then dissolved at the concentration of 2 mg/mL. The standard lead solution was diluted with deionized water until a final concentration of 10 μg/mL was reached. Then equal volumes of polysaccharide sample and lead solution were mixed with agitation at 160 r/min for 3 h at room temperature. Alcohol (four volumes) was added to the mixture, which was then left at room temperature. One hour was allowed for precipitation of the polysaccharides to take place. Subsequently, the mixture was centrifuged (9,000 r/min, 10 min) to precipitate the polysaccharides. The supernatant was then used for determination of the lead concentration by Atomic Fluorescence Spectroscopy (AFS). Deionized water was used instead of polysaccharide as the control group.

Calculation of the clearance ratio was performed as shown below:


$$\begin{array}{l} \text{Clearance ratio }\left(\text{\%}\right)=\frac{\text{Difference in Pb concentration between control group and polysaccharide}-\text{treated group}}{\text{Pb Concentration in control group}}\times 100\text{\%} \end{array}$$

### 2.4. Determination of molecular weight and analysis of *N*-terminal and inner amino acid sequences of APL

For molecular weight measurement, protein standards with molecular weights 669,000, 440,000, 158,000, 75,000, 44,000, 29,000, and 13,700, 225,000, respectively were utilized for calibrating the Superdex 200 10/300 column. The molecular weight of APL was estimated from the calibration curve plotting elution volume against the log of molecular weight.

Purified APL was subjected to sodium dodecyl sulfate-polyacrylamide gel electrophoresis (SDS-PAGE). The band was cut out from the gel stained with Coomassie blue. The bands on the SDS-PAGE gel and PVDF membrane were recovered and used for partial amino acid sequence analysis and N terminal sequence analysis, respectively. The BLAST/NCBI database was used for sequence homology search.

### 2.5. Analysis of FT-IR spectrum and monosaccharide composition of APL

The spectrum of APL was obtained by employing a Nicolet iS5 Fourier transform infrared spectrophotometer in the wave number range of 4,000–400 cm^−1^ following pulverization of the sample with KBr powder and compression into pellets.

The procedures for determination of monosaccharide composition of APL were as described by Zhao (21): APL (2.0 mg) was dissolved in 2 mL 4 M trifluoroacetic acid in an ampoule, sealed under N_2_, put in a 110°C oil bath for 8 h, and cooled down to room temperature before centrifugation. The supernatant collected was neutralized. Hydrolyzed APL and monosaccharide standards were dissolved in 0.3 M NaOH before a 0.5 M methanol solution of 1-phenyl-3-methyl-5-pyrazolone (PMP) was added. Fucose was added as an internal standard before derivatization using PMP. Reaction was allowed to go on for 30 min at 70°C. The mixture was cooled down to room temperature and neutralized, dissolved in CHCl_3_, vigorously agitated, centrifuged and the organic phase underneath the aqueous layer was disposed of. Extraction was repeated three times. The aqueous layer was filtered through a 0.45-μm membrane and diluted with water before HPLC on a C18 column using a wavelength of 250 nm for detection of UV. The mobile phase employed was 50 mM sodium phosphate (pH 6.9) with (A) 15% and (B) 40% acetonitrile, using a gradient elution of 0–8–20% buffer B with a linear increase from 0–10–30 min.

### 2.6. Detoxifying effect of APL to mitigate lead poisoning in rats

#### 2.6.1. Animals

Male Sprague-Dawley rats (250–280 g) were acclimatized for 7 days prior to the experiment. The protocol used has been reviewed and endorsed by the Animal Ethical and Welfare Committee.

#### 2.6.2. Preparation of solutions used for chronic lead poisoning

Pb(Ac)_2_ solution (4 mg/mL) was employed to establish the lead poisoning model. EDTA-2NaCa (37.5 mg/mL) served as a positive control by virtue of its ferrous ion chelating activity. Pentobarbital sodium solution (3%) was employed to anesthetize the animals.

#### 2.6.3. Lead clearance effect of APL for mitigating chronic poisoning in rats

The model of acute lead poisoning is established by giving experimental animals a single administration of a lead compound (27) whereas establishment of the model of chronic lead poisoning involves multiple administrations of a lead compound (28).Rats were divided in random to groups (five rats/group). The rat model of chronic lead poisoning was established by daily intragastric Pb(Ac)_2_ injections at a high dosage of 20 mg/kg body weight for 1 week, and also at a low dosage (5 mg/kg body weight) of Pb(Ac)_2_ solution for 30 days after a recovery period of 3 days. The unpoisoned control group (CON) received injections of the same amount of normal saline. APL was intragastrically administered at three dosages (40, 80, and 160 mg/kg body weight), daily for 30 days. The various groups received different treatments as shown in Table 1 below.

**Table 1:** Various treatments of rats in the chronic lead poisoning study for investigating the protective actions of EDTA(positive control) and various dosages of APL.

**Group**	**Chronic poisoning**	**Dosages and treatments**
CON	No	Intragastric administration of saline (~2 mL)
MOD	Yes	Intragastric administration of saline (~2mL)
EDTA	Yes	Intragastric administration of EDTA with 300 mg/kgBW
APL-L	Yes	Intragastric administration of APL with 40 mg/kgBW
APL-M	Yes	Intragastric administration of APL with 80 mg/kgBW
APL-H	Yes	Intragastric administration of APL with 160 mg/kgBW

#### 2.6.4. Analysis of residual lead in blood and tissue samples

Blood samples were collected from the rat eye socket vein every 6 days i.e., on day 0, day 6, day 12, day 18, day 24, and day 30. Blood samples were collected and stored at −20°C. Following the final blood collection, rats were over-anesthestized to death, and the heart, kidneys, liver, and spleen were collected, lyophilized and ground into powder. Some liver tissues were immediately stored at −80°C for proteomic analyses. Serum samples were prepared from the last batch of blood samples for the analysis of ALT, AST, CAT, SOD enzyme activities and determination of MDA levels. The organ powders were acidolyzed by using HCl-HNO_3_ solution (v/v 3:1), and the lead concentrations were determined using Atomic Fluorescence Spectroscopy.

#### 2.6.5. Protein preparation for proteomic analyses

Based on the results of analysis of lead residues in various organs, the liver samples of CON, MOD, and APL-H treated groups were prepared and dispatched to Biomarker Technologies for further proteomic analyses. After the pretreatment of the liver powder samples, the proteins were labeled with TMT reagent and peptides were identified by nano UPLC-MS/MS technology.

### 2.7. Statistical analysis

Data from experiments described above in 2.6.3 and 2.6.4 were subjected to ANOVA. The resulting MS/MS data were processed using Proteome Discoverer 1.4. According to the *p*-value of the primary data, data with *p* ≤ 0.05 and difference ratio ≥1.2 were selected for further analysis. The KEGG pathway enrichment analysis of the differentially expressed proteins were performed and visualized as enrichment map networks using ClusterProfiler (version 4.2.2).

## 3. Results

### 3.1. Purification of APL with lead-clearance activity

**Figure 1:**
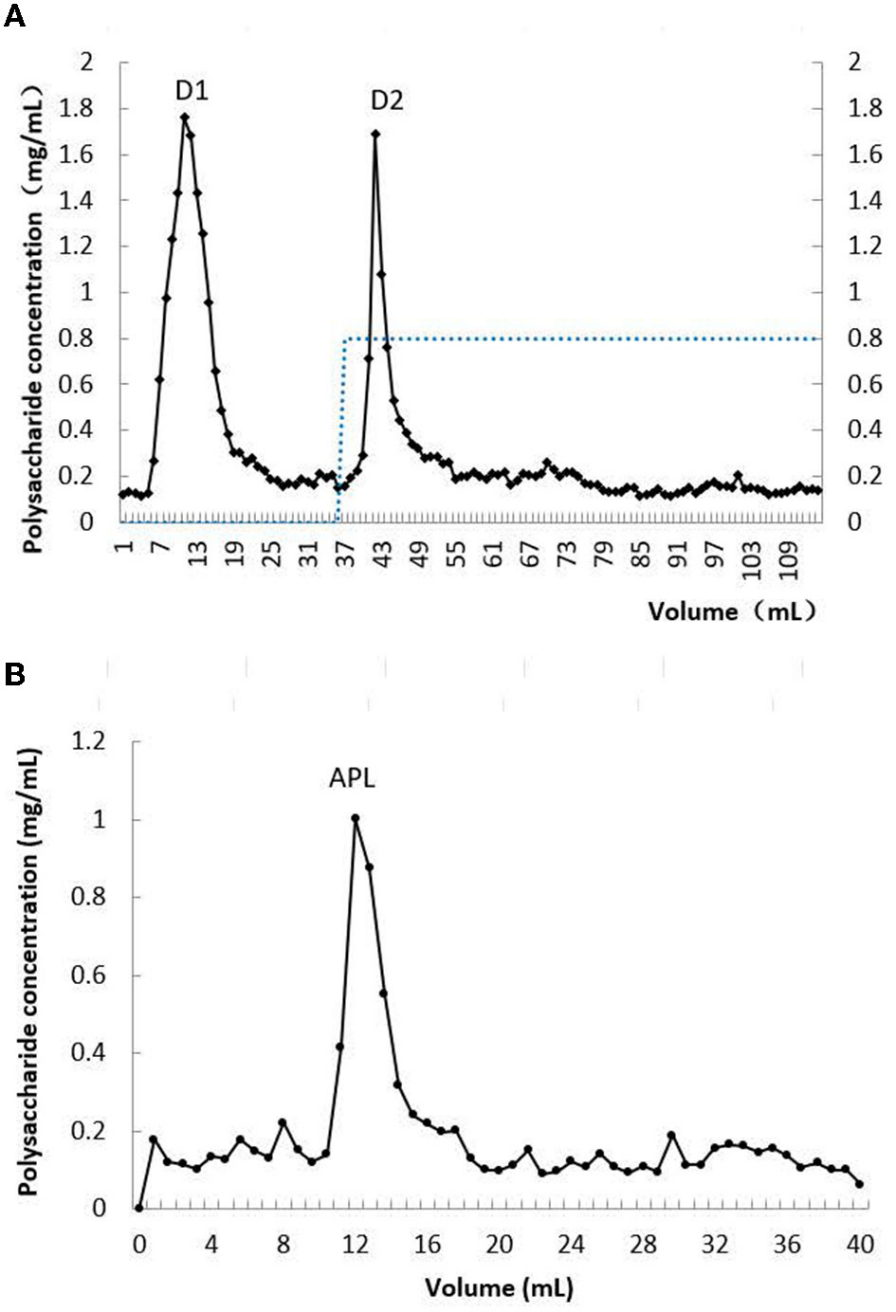
Chromatographs of crude extract of *A. polytricha*. **(A)** Profile of elution from DEAE-cellulose column; **(B)** profile of elution from Sephadex 200 column.

After hot water extraction and precipitation with ethanol, a crude carbohydrate-containing extract with lead-clearing activity was prepared from *A. polytricha* fruiting bodies. The crude extract was sequentially fractionated on DE-52. Two peaks with polysaccharide content were eluted with distilled water and 0.8 M NaCl solution, respectively (Figure 1A). The fractions eluted with 0.8 M NaCl solution exhibited lead-clearance activity as determined *in vitro*. The active peak was then chromatographed on Superdex 200. Only one peak designated as APL was eluted. Its molecular mass was judged, based on its elution volume, compared with those of protein standards, to be 252 kDa (Figure 1B). This APL peak was composed of predominantly protein and only a minor amount of polysaccharide, with a high protein: polysaccharide ratio of 86.7:1. Thus APL was a glycoprotein.

### 3.2. Characterization of APL

#### 3.2.1. N-terminal amino acid and internal peptide sequence analysis

Edman degradation method revealed that the *N*-terminal amino acid sequence of APL was HDDMGMSAMM. The internal amino acid sequences of APL were unraveled by ESI-MS/MS. Based on the searching of results in the BLAST/NCBI database, there were 12,622 amino acid sequences in APL similar to other protein or peptide sequences because of its high protein content. In most of the sequences, APL manifested pronounced homology to ClpB ATP protease from the fungus *Paracoccidioides brasiliensis*, with 13 identical peptides sequences and a coverage ratio of 23.48%. Other peptide sequences, including LLDQGQAGDNVGLLLR, HYAHVDCPGHADYVK, AYDQIDAAPEEK, GYRPQFYFR and TVGAGVVAK, demonstrated marked resemblance to elongation factor Tu from the bioluminescent fungus *Mycena chlorophos*, and the coverage ratio was 15.4%. These results of sequence analysis corroborated the presence of peptide in APL.

#### 3.2.2. Monosaccharide composition and FT-IR spectroscopy analysis

The monosaccharide composition of APL was examined with HPLC following the PMP derivatization method, and the monosaccharide content in APL is listed in Table 2. APL was composed of Man, Rha, Glu, Gal, Xyl, glucuronic acid and galacturonic acid in the ratio of 27.8:8:19.3:22.7:8.7:30:9.

**Table 2:** Content of various monosaccharides in APL.

	**Man**	**Rha**	**Glu**	**Gal**	**Xyl**	**GluA**	**GalA**
Content (‰)	0.501	0.131	0.347	0.408	0.130	0.582	0.175

**Figure 2:**
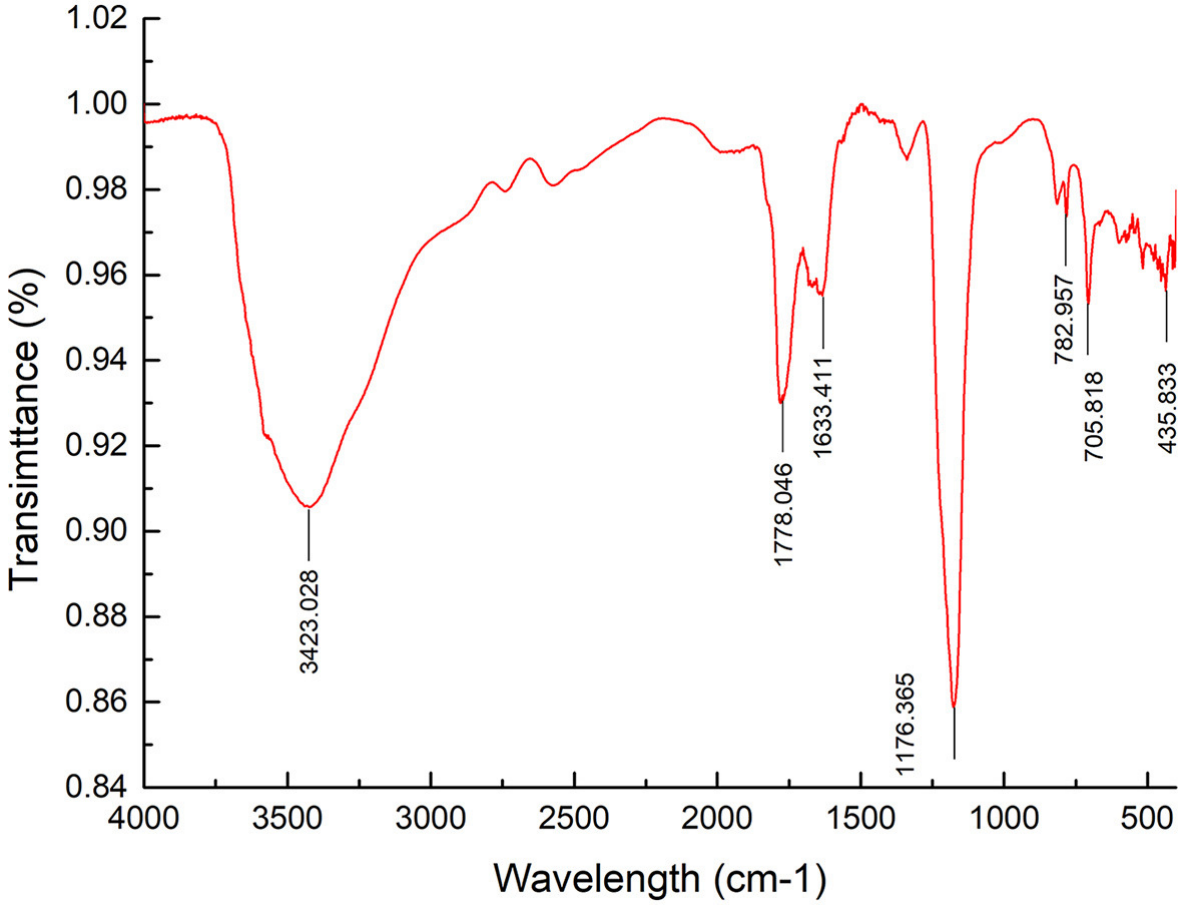
FT–IR spectrum of *A. polytricha* glycoprotein APL.

FT–IR spectrum of APL G was recorded from 4,000 to 400 cm^−1^ (Figure 2). The FT-IR spectrum illustrated a strong and wide stretch vibration of O–H at 3,423 cm^−1^. The peak at 1,778 cm^−1^ was attributed to C=O valent vibration of the O-acetyl group, while the peak at 1,633 cm^−1^ was caused by asymmetric and symmetric stretching vibrations of carboxylic acid groups. The stretching peaks at 1,176 cm^−1^ reflected the existence of C–O bonds and the pyranose form of sugar. These results further confirmed the presence of polysaccharide in APL. In addition, there were some small peaks in the range of 700 cm^−1^ to 800 cm^−1^, indicating the presence of single or double substituent group on the benzene ring.

The above analysis verified that APL was a novel glycoprotein, which was different from previously reported glycoproteins. The characteristics of the glycoprotein from *A. polytricha* are shown in Supplementary Table S1.

### 3.3. Protective effects of APL in protecting from chronic lead toxicity *in vivo*

#### 3.3.1. Signs of lead toxicity in rats

**Figure 3:**
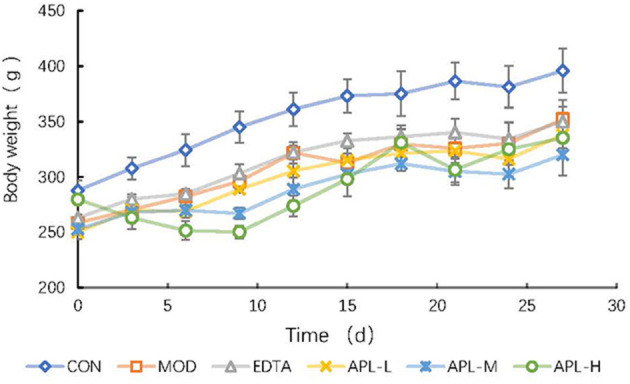
Time course study of the effect of APL in preventing lead-induced fall in body weight. APL-L, APL-M, and APL-H represent 40, 80, and 160 mg APL/kg body weight, respectively. Results are presented in mean ± standard deviation (*N* = 5/group).

To establish a rat model of chronic poisoning, Pb(Ac)_2_ at a high dosage was considered as an acute stimulation, and a low dosage was injected daily for maintenance. Rats in the CON group exhibited normal mental performance and had neat fur, but rats in the lead exposure group were characterized by a marked reduction of food consumption, physical weakness, lethargy, an emaciated appearance, and disorderly fur. The body weight measurements showed that the growth rate of the lead exposed group lagged behind and was only 46.04% of that of the CON group on the first 7 days of the lead stimulation period (Figure 3). The adverse effects reflected the toxic influence of lead on rats. Concurrent administration to that of EDTA, which increased body weight by 8.3% compared with that of the MOD group.

#### 3.3.2. Protective effect of APL on lead concentration in whole blood and serum enzymes

**Figure 4:**
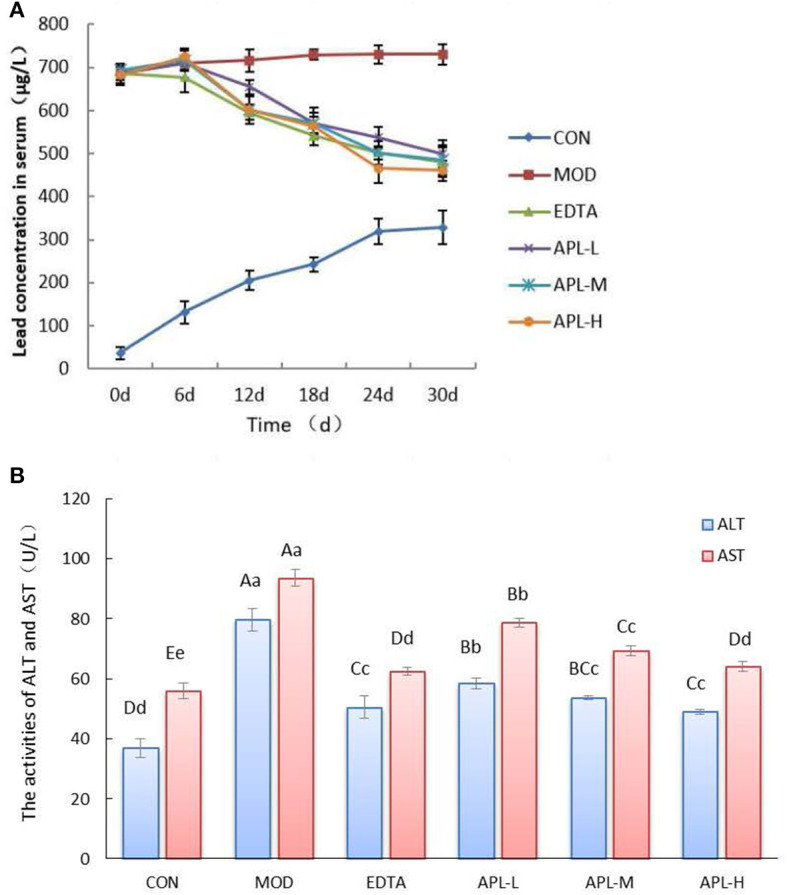
APL-elicited inhibition of rise in serum biochemical indexes in different experimental groups following lead administration. **(A)** Time course of serum lead concentration; **(B)** Serum enzyme (ALT and AST) activities. APL-L, APL-M, and APL-H represent 40, 80, and 160 mg APL/kg body weight, respectively. Results are presented in mean ± standard deviation (*N* = 5/group). a, b, c indicated the significant difference (*p* < 0.05), A, B, C indicated the significa.nt difference (*p* < 0.05).

Compared with the CON group, the blood lead level in chronic toxicity groups increased 18.6 fold prior to APL administration (Figure 4A). In APL-treated groups, the blood lead levels declined as the administration time was extended. By the 30th day, there was a statistically significant difference in blood lead concentration between the model group and APL-treated groups. The blood lead levels in groups treated with the three dosages of APL were reduced vs. the MOD group. This was similar to the reduction observed in the EDTA group compared with the MOD group, suggesting that APL could act like EDTA, to promote lead elimination from blood. The blood lead concentrations in groups treated with high, medium and low dosages of APL were finally lowered by 36.9%, 33.6%, and 31.6%, respectively.

In addition, serum activities of ALT and AST in the model group were significantly elevated vs. the CON group (*p* < 0.01), as shown in Figure 4B. However, the activities of both enzymes (ALT and AST) in APL treated groups were significantly diminished compared with those of the MOD group (*p* < 0.01), although they still remained elevated in comparison with the CON group. The results indicated that lead induced liver tissue injury, and APL exerted a protective effect on the liver to combat lead toxicity. However, the serum oxidative stress indicators in different APL-treated groups were not significantly changed (data not shown).

#### 3.3.3. Effects of APL on promoting lead elimination from the liver and kidney

The rat organs were tested for accumulation of lead. The results disclosed that the injected lead mainly accumulated in hepatic and renal tissues, as shown in Table 3. The lead contents of liver and kidney in the MOD group were elevated 10.6 and 10.3 fold, respectively. Compared with the MOD group, interventions with the three dosages of APL significantly (*p* < 0.05) lowered the hepatic and renal lead contents, and the clearance effect was dependent on the dosage of APL employed. By the 30th day, the clearance ratio of liver and kidney at the high APL dosage of 160 mg/kg body weight were 44.5% and 18.1%, respectively. The findings demonstrated that APL administration could promote lead disposal from the liver and kidneys, The effect was especially remarkable in the liver. These results indicated that oral intake of APL was highly efficacious in protecting from lead-induced liver injury, with a superior effect compared with intervention using EDTA.

**Table 3:** APL-elicited inhibition of rise in lead concentration and increase of lead clearance ratio in the liver and kidneys following lead administration.

	**Liver**		**Kidney**	
	**Lead concentration (**μ**g/g)**	**Lead clearance ratio (%)**	**Lead concentration (**μ**g/g)**	**Lead clearance ratio (%)**
CON	8.1 ± 2.8^Ee^	–	6.8 ± 1.5^Dd^	–
MOD	86.2 ± 3.2^Aa^	0	70.3 ± 5.8^Aa^	0
EDTA	60.3 ± 4.1^Cc^	30.1	30.9 ± 2.7^Cc^	56.1
APL-L	67.4 ± 0.8^Bb^	21.9	64.3 ± 4.3^ABa^	8.5
APL-M	63.6 ± 2.1^BCc^	26.2	58.4 ± 2.8^Bb^	16.9
APL-H	47.8 ± 4.6^Dd^	44.5	57.6 ± 1.1^Bb^	18.1

APL-L, APL-M, and APL-H represent 40, 80, and 160 mg APL/kg body weight, respectively. Results are presented in mean ± standard deviation (N = 5/group). a, b, c indicated the significant difference (p < 0.05), A, B, C indicated the significant difference (p < 0.05).

#### 3.3.4. Differentially expressed proteins in the liver of APL intervention exposed to lead

**Figure 5:**
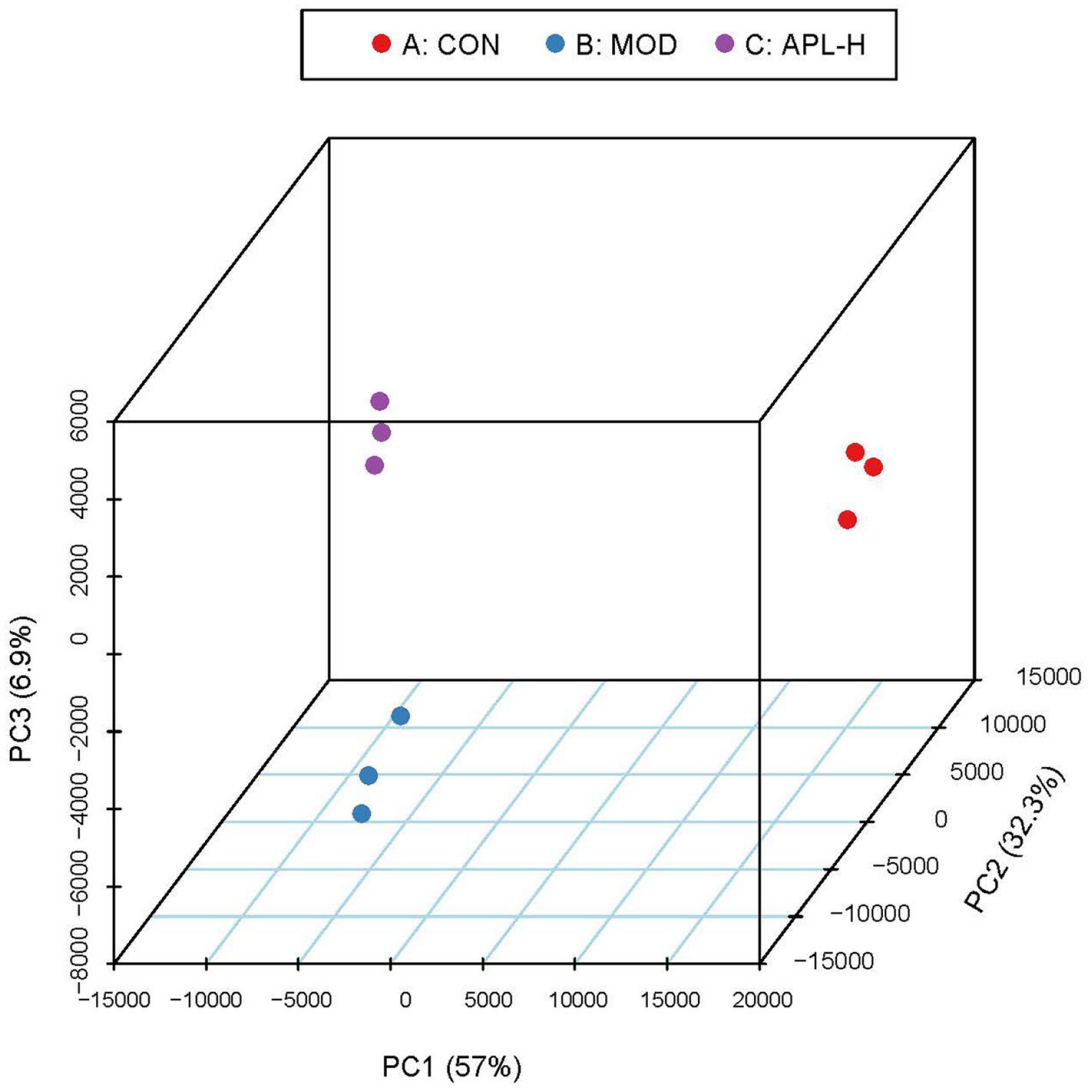
PCA score scatter plots of identified proteins.

**Figure 6:**
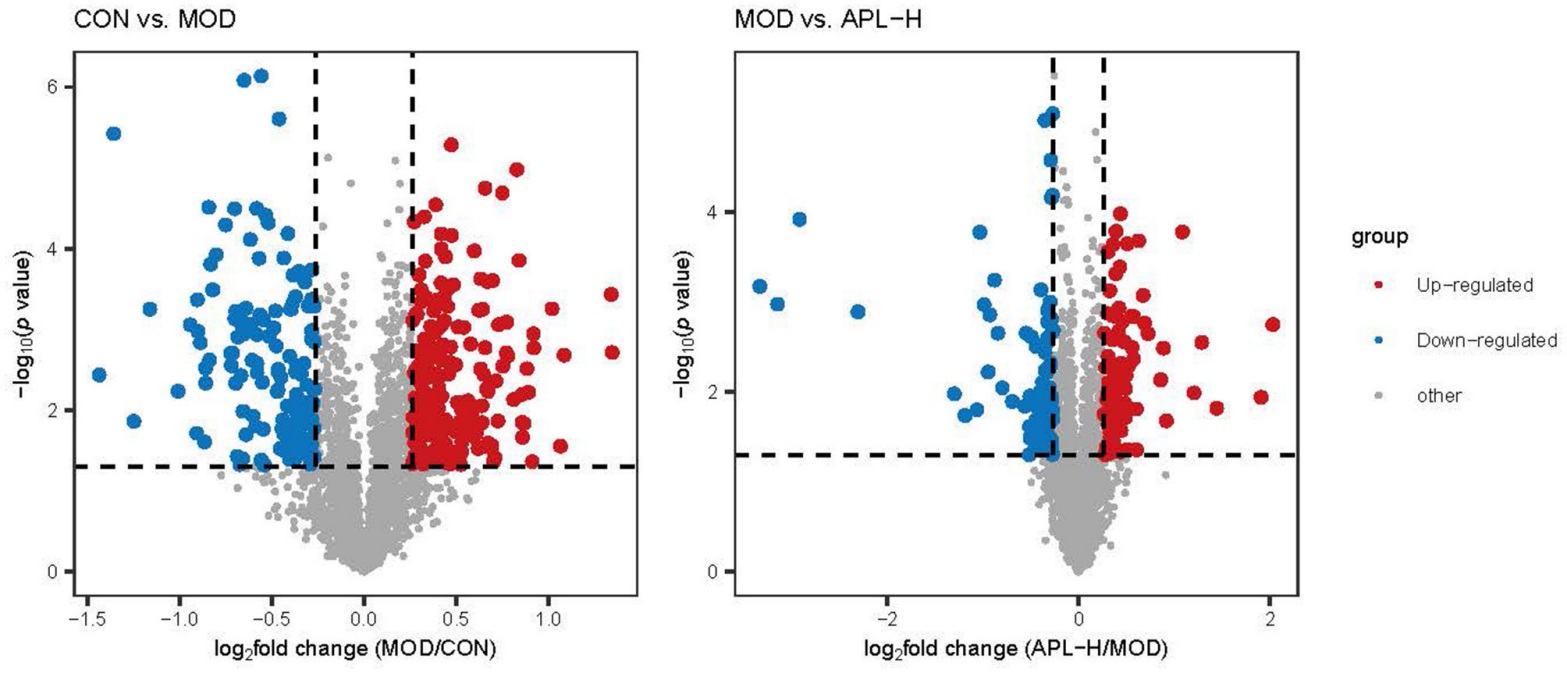
Volcano plots of DEPs between different groups.

To ascertain the protective mechanisms of APL on lead-elicited hepatic injury in rats, liver tissues were analyzed with the proteomic technique. TMT labeling quantitative method was used to ascertain the differentially expressed proteins in the livers of animals in the CON, MOD and APL-H treated groups. The PCA displays the distribution and statistical significance of the identified proteins in Figure 5. Under the experimental conditions utilized, 2,541 proteins were detected, among which 351 differentially expressed proteins (DEPs) comprising 209 upregulated and 142 downregulated were identified in the MOD group, compared to the CON group. Totally 156 DEPs were screened between the APL-H group and MOD group. Eighty DEPs were up-regulated whereas 76 DEPs were down-regulated. Volcano graphs were used to demonstrate the distribution of DEPs (Figure 6). The screening criteria for DEPs were as follows: fold change ≥1.2 or ≤ -1.2; *p* < 0.05 by ANOVA. Of these DEPs, the expression levels of 41 proteins, which were involved in many important biological functions including immune system process, vesicle-mediated transport, defense response, intracellular protein transport, cytoskeleton organization, nucleobase-containing small molecule metabolic process, carbohydrate and derivative metabolic process and aging, were normalized after treatment with APL-H. To create an overview, Venn diagrams including DEPs between different groups were generated, heatmap and the details and of normalized proteins by APL-H treatment are shown in Supplementary Figure S1, Supplementary Table S2.

#### 3.3.5. Bioinformatic functional analysis of DEPs

**Figure 7:**
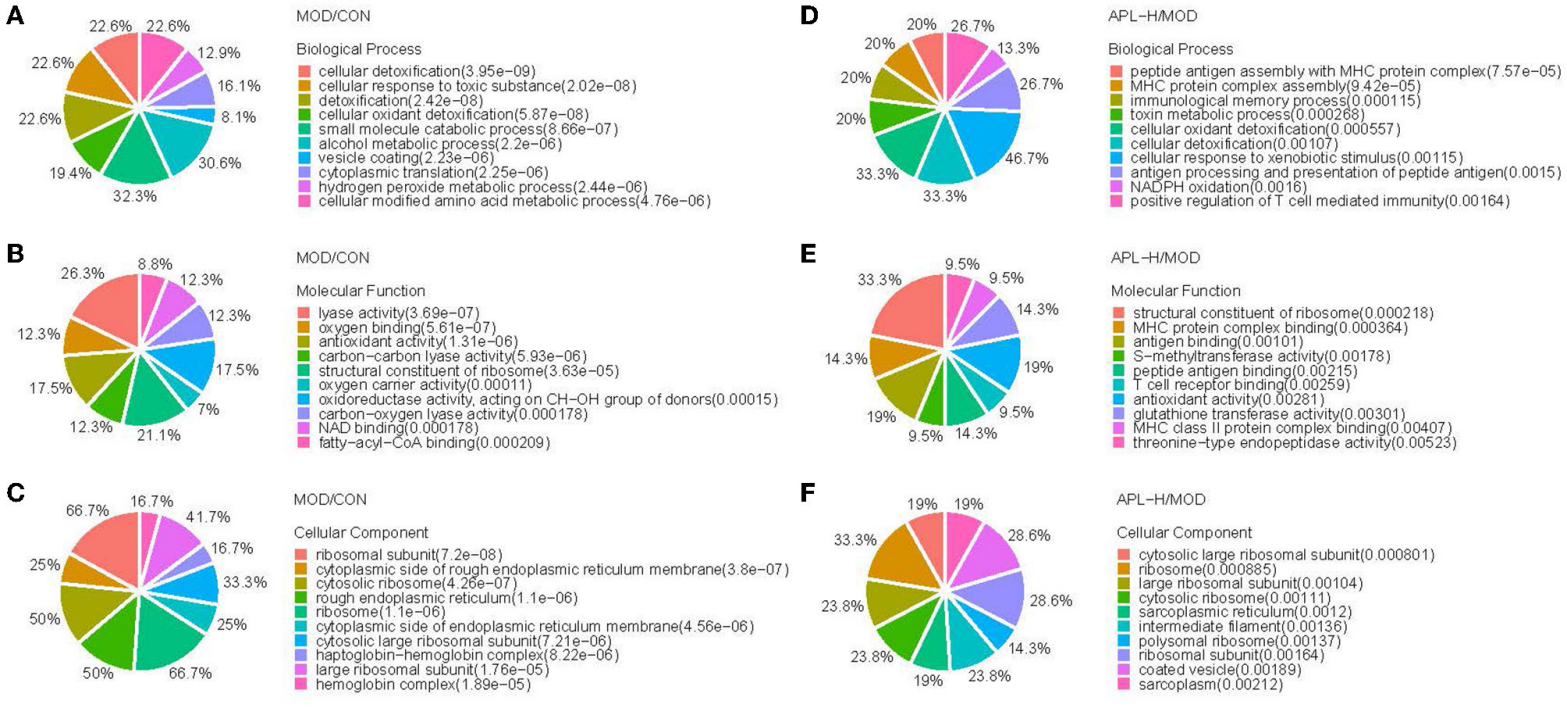
GO analysis of the differentially expressed proteins. **(A)** Biological process analysis of MOD/CON; **(B)** Molecular function analysis of MOD/CON; **(C)** Cellular component analysis of MOD/CON; **(D)** Biological process analysis of APL-H/MOD; **(E)** Molecular function analysis of APL-H/MOD; **(F)** Cellular component analysis of APL-H/MOD.

**Figure 8:**
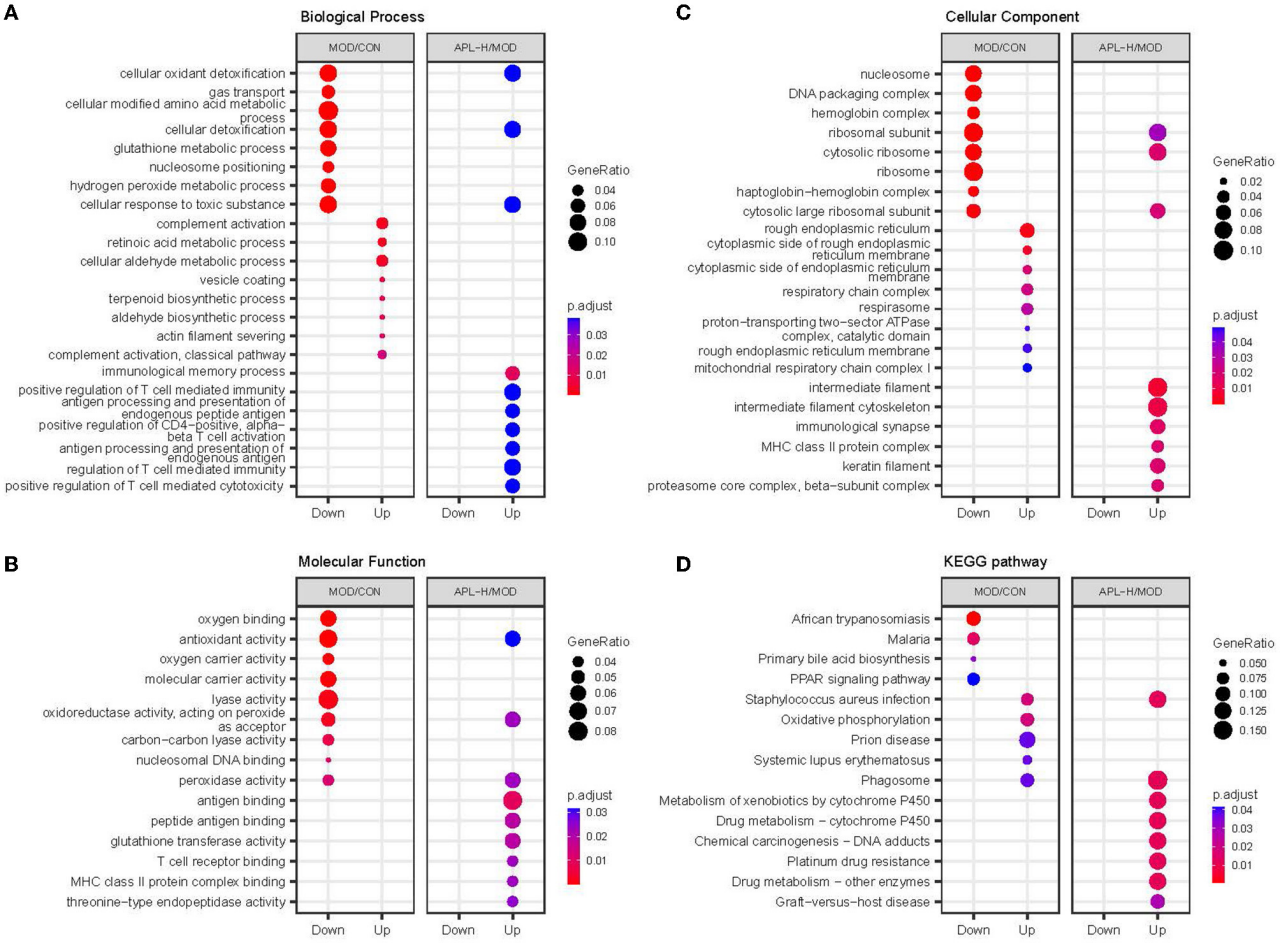
GO enrichment and KEGG pathway analysis of different groups. **(A)** Biological process analysis; **(B)** Molecular function analysis; **(C)** Cellular component analysis; **(D)** KEGG pathway analysis.

GO enrichment analysis was carried out in pairs of the MOD group and CON group, APL-H group and MOD group. The analysis showed the characterized 346 DEPs between CON and MOD groups. Among these DEPs, 250 proteins were annotated for their biological processes (BP), 253 proteins were annotated for their molecular functions (MF), and 259 proteins were annotated for their cellular component (CC). The top 10 categories were calculated based on the protein counts and are shown in Figures 7A–C. The upregulated proteins in the MOD group were significantly enriched in complement activation, small molecule metabolic and biosynthetic processes, and actin filament severing, while the downregulated proteins were significantly enriched in processes associated with detoxification and response to toxic substance correlation, cellular modified amino acid metabolism, and hydrogen peroxide metabolism (Figure 8). For the analysis of BP, MF and CC of DEPs exposed to lead, the majority of proteins obtained were involved in processes associated with small molecule catabolism (32.3%), alcohol metabolism (30.6%), cellular detoxification (22.6%), and cellular response to toxic substance (22.6%) in BP, while lyase activity (26.3%), structural constituent of ribosome (21.1%), antioxidant activity (17.5%), and oxidoreductase activity, acted on CH-OH group of donors (17.5%) in MF. GO analysis of DEPs was consistent with the known pathophysiologic mechanism of lead induction of liver injury. For the analysis of BP of proteins normalized by APL-H treatment were cellular response to xenobiotic stimulus (46.7%), cellular detoxification (33.3%), cellular oxidant detoxification (33.3%), and positive regulation of T cell mediated immunity (26.7%). In terms of MF, the results of normalized proteins were involved in structural constituent of ribosome (33.3%), antioxidant activity (19%), antigen binding (19%), and MHC protein complex binding (14.3%) (Figures 7D–F). Normalized protein enrichment results revealed that cellular detoxification function was elevated by APL-H associated with improvements in immunity and antioxidant function.

**Figure 9:**
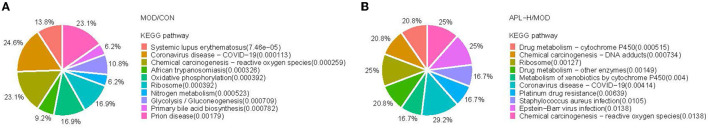
KEGG analysis of the differentially expressed proteins. **(A)** MOD/CON; **(B)** APL-H/MOD.

Based on 351 DEPs between CON group and MOD group, the KEGG pathway analysis showed that 166 enriched DEPs identified 41 significantly accumulated pathways (Figure 8D). Compared to the CON group, the DEPs in the MOD group were significantly enriched in systemic immune diseases, chemical carcinogenesis-reactive oxygen species, oxidative phosphorylation, and the top 10 pathways were calculated based on the protein counts and are illustrated in Figure 9A. 62 DEPs in APL-H group were enriched in 28 accumulated pathways based on the KEGG analysis. These proteins are mainly involved in virus infection, chemical carcinogenesis, drug resistance and metabolism, and the top 10 categories are shown in Figure 9B.

**Figure 10:**
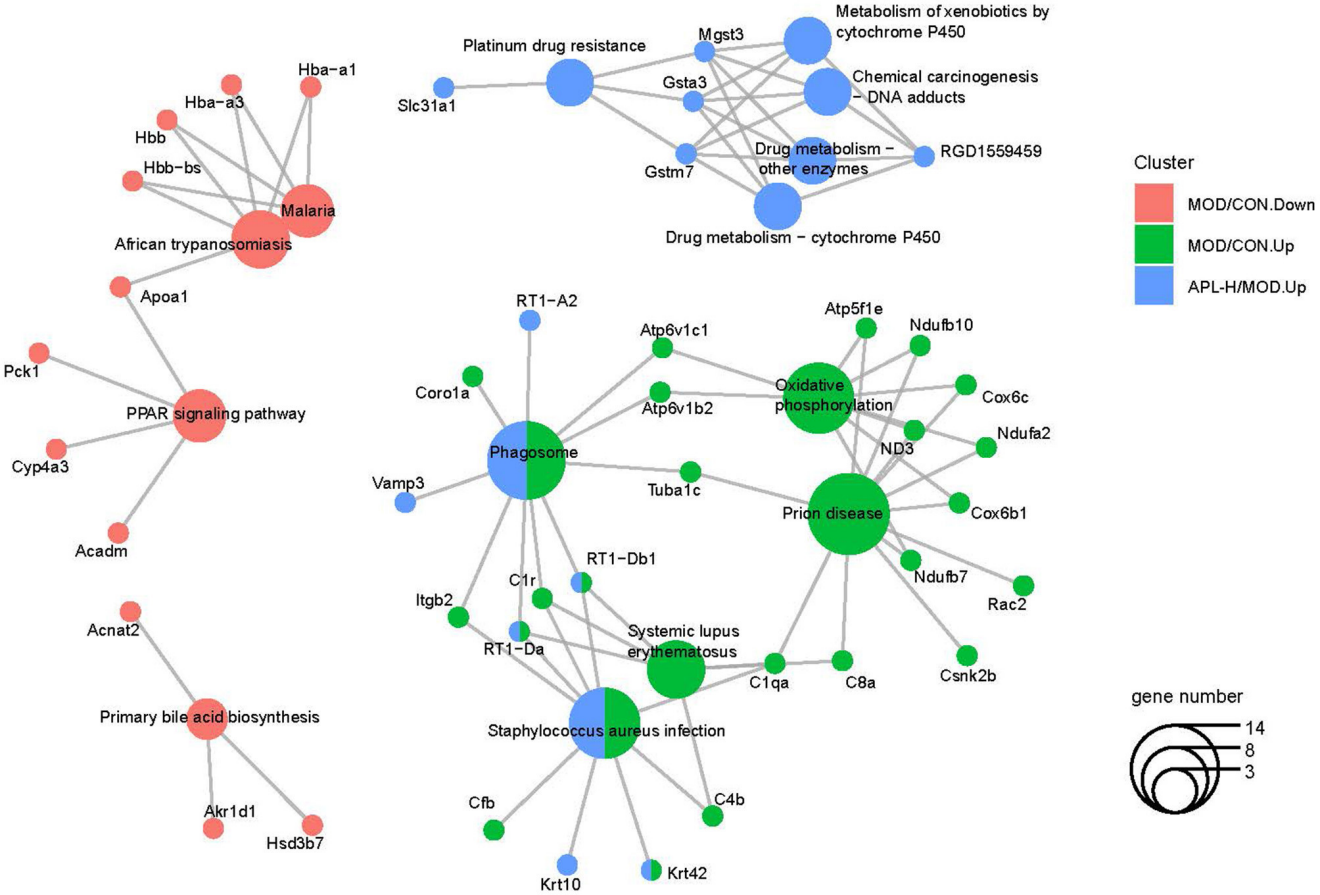
Functionally grouped networks with pathways and genes derived from the Cytoscape database.

To acquire a better understanding of all the pathways involved, enrichment analysis was conducted with ClusterProfiler software. The function annotation of lead-triggered genes and APL-H normalized genes are illustrated in Figure 10, the gene network analysis emphasized on 42 genes and 14 pathways. Enrichment analysis of the pathways disclosed that certain pivotal genes were highlighted in the functionally grouped network. They are crucial genes that connect the enriched metabolic pathways and are found at the node of the network. In Figure 10, *Mgst3, Gsta3, Gsta7* are the important genes that connect five metabolic-related pathways, comprising drug metabolism-cytochrome P450, drug metabolism-other enzymes, chemical carcinogenesis-DNA adducts, metabolism of xenobiotics by cytochrome P450 and platinum drug resistance. Likewise, *RT1-Db1, C1r, RT1-Da* are the key genes that link three metabolic-related pathways, including phagosome, systemic lupus erythematosus, and *Staphylococcus aureus* infection. Furthermore, enrichment analysis revealed that all 14 pathways are key pathways associated with hepatoxicity brought about by lead exposure.

## 4. Discussion

Numerous investigations have disclosed the toxic action of lead ensuing in a diversity of pathological and clinical outcomes in virtually all of the organs, including, hepatic and renal injury, inflammation and immunomodulation, often accompanied by anemia, high blood pressure, and heart diseases (29–32). At present, chelating drugs are the most common therapeutic agents utilized to eliminate heavy metals and restore metabolic imbalance, albeit with undesirable side effects (33, 34). Metal chelators exhibit shortcomings including neurotoxicity caused by redistribution of heavy metals from tissues to the brain, loss of important metals including copper, calcium and zinc due to non-specific chelation and toxicity to the liver. The chelating drugs comprise penicillamine (penicillin derivative), dimercaprol, succimer (dimercaprol derivative) and sodium calcium edetate, all with a host of possible untoward side effects such as allergy, hypocalcemia, cardiac arrhythmias, respiratory failure, fever, headache, nausea, and vomiting (35). The U.S. Food and Drug Administration discourages consumers from buying over-the-counter chelation or detoxification products. FDA-approved chelating agents have to be used with caution under medical supervision. Hence natural antidotes to deal with heavy metal poisoning is preferable to metal chelators (36).

Lead exposure ensues in organ damage because of toxicity caused by oxidative stress and impairment of the antioxidant defense system. Damage to lipids, proteins, and nucleic acids is involved (37). Some studies reported that food nutraceuticals not only have metal chelating properties and antioxidant activity but are also implicated in metabolism of metals (38). Algae, curry leaf, garlic, ginger, grape, green tea, tomato, probiotics, anthocyanins, naringin, curcumin, puerarin, gamma oryzanol, and quercetin have also been reported to alleviate some aspects of lead toxicity (39). Recent studies have demonstrated that antioxidants and free radical scavengers are useful in protecting against lead toxicity (9, 40–42), but the exact mechanism of the detoxifying effect toward the liver still awaits elucidation.

A crude extract of the mushroom *Pleurotus tuber regium* mitigated arsenic-chromium toxicity as evidenced by increased feed consumption, reduced activities of aminotransferases ALT and AST in the liver and decreased hepatic and renal malondialdehyde in rats (43).The detoxifying action of *P. tuber regium* toward arsenic-chromium was similar to the action of *A. polytricha* toward lead (43).The present report represents a pioneering study on the protective action of a mushroom protein against lead-elicited organ damage. It has been reported that *A. polytricha* could serve as a copper biosorbent (22). *A. polytricha* fruiting bodies were used for removing Cr, Cu, Hg, Zn and Cd (23, 44, 45). Nevertheless, a purified compound with unique molecular property has not yet been isolated. In this study, a glycoprotein APL with a high protein and a low carbohydrate content was purified and characterized from *A. polytricha* fruiting bodies, and further shown to be efficacious for removing lead and protecting from lead-elicited toxicity in rats with an immunoregulatory mechanism. Epidemiological and experimental studies have demonstrated lead-triggered immunotoxicity with an impact on cellular as well as humoral immune response. The function of Th cells is affected and the risk of the developing hypersensitivity and autoimmunity is heightened (46). APL combated the immunotoxicity of lead by immunoregulatory mechanisms. Previously only polysaccharides (17–20) but not proteins have been isolated from *A. polytricha*. Although APL displays homology in amino acid sequences of internal peptides with elongation factor Tu from the bioluminescent fungus *Mycena chlorophos* and the ClpB ATP protease from the fungus *Paracoccidioides brasiliensis*, elongation factor Tu from fungi and bacteria possess molecular weights in the vicinity of 50 kDa (47) and ClpB ATP protease from the fungus *Paracoccidioides brasiliensis* has a size of 100 kDa (48), both much smaller than that of APL (252 kDa) and neither of them has been reported to contain a carbohydrate moiety. Hence APL is a novel protein and the findings reported in the present article would be a useful addition to the existing literature. The vast majority of the currently available fourteen papers on *Mycena chlorophos* are about bioluminescence of the fungus including the bioluminescent principle hispidin (49) and 3-hydroxylase (50).

Based on the assays in this study, the overall findings showed that administration of graded doses of APL attenuated the manifestations of lead toxicity, removed lead from the serum and organs, especially the liver and kidneys, and reinstated the hepatic function biomarkers to near normality. In the present investigation, lead inhibited body growth, and the excess lead accumulated in the blood, liver and kidneys (Figures 3, 4 and Table 3). Compared with the CON group, the body weights of rats underwent a significant decline in response to lead administration, whereas lead accumulation in the blood and organs rose significantly (*P* < 0.05). Lead-induced toxicity was also displayed in the dispirited mental performance and dull fur appearance. The clinical signs and biochemical indexes suggested that the rat model can serve as a useful model of lead-induced toxicity for investigation.

EDTA as a chelant to adsorb heavy metals was used as a positive control in this study. The results showed that lead accumulation in the blood, kidneys and liver was abated after administration of EDTA and APL vs. the MOD group. There was a statistically significant (*P* < 0.01) difference in the hepatic lead content between groups treated with different dosages of APL and the MOD group, which implied a dose-dependent bioactivity for lead elimination. The high dose of APL outperformed the dose of EDTA employed in this study, signifying that the toxic and untoward effects of lead could be minimized by APL.

ALT and AST are regarded as hepatic marker enzymes which exist mainly in the mitochondrion and cytoplasm, and are released into the blood plasma following liver injury or damage (51, 52). In this study, the serum activities of AST and ALT were significantly (*P* < 0.05) escalated in the MOD group, compared to the CON group (Figure 3B), reminiscent of the findings of Gao (42). Lead is known to exert hepatotoxicity in rats and results in liver cell damage due to oxidative stress. The elevated activities of liver enzymes indicate hepatic tissue damage as an outcome of disruption of liver cell membrane intactness and necrosis culminating in enhanced membrane permeability and leakage and liberation of these enzymes into the blood. After administering APL over a period of 30 days, there was a significant lowering (*P* < 0.05) of AST and ALT activities, in all of the APL-treated groups (treatments with 40, 80, and 160 mg/kg APL) compared to the MOD group. The degree of reduction brought about by 160 mg/kg APL treatment was similar to that achieved by treatment with 300 mg/kg EDTA. These findings revealed APL could prevent and protect the liver from lead-elicited damage with a higher efficacy compared with EDTA. Oral intake of the antioxidant vitamins C and E inhibited the rise in activities of the liver aminotransferases in the liver and the blood circulation following lead intoxication (53).

The liver is the body's most extensive organ essential for detoxification and immune function, and thus it was selected as a target organ for studying the mechanism of APL intervention. Our intent was to explore the specific cellular response and biological process following lead hepatoxicity and APL treatment. All proteins in the liver were examined for quantitative analysis. Bioinformatics analysis showed that prominent clusters for cellular detoxification were downregulated in the MOD group, for example, Parkinson disease protein 7 homolog, thioredoxin, glutathione peroxidase 1, superoxide dismutase and hemoglobin subunit (54–58) were all decreased in abundance in the MOD group. These downregulated proteins would lead to inhibited repairment of methylglyoxal- and glyoxal-glycated proteins and release of repaired proteins, reduced the functions of nucleotide repair system and cell protection against oxidative stress and metal toxicity, triggered the response to intracellular nitric oxide, enhanced caspase-3 activity and induced apoptosis and cell death (58). On top of detoxification, evidence exists at the pathway level that biomolecular synthetic and metabolic processes, cytoskeletal filament and cytosolic ribosome component, drug metabolism, and energy homeostasis were impacted differently upon exposure to lead, in keeping with other reports on the effects of poison in the mammalian liver (59). In the present study, APL was able to mitigate lead toxicity as judged by the decline in the circulatory and hepatic as well as renal lead concentrations increased due to lead exposure. This is because the liver and the kidneys are involved in phase 1, phase 2 and phase 3 of xenobiotic detoxification. In humans with chronic exposure to lead, plasma lead level and liver function enzymatic markers were elevated and data on serum creatinine and urea revealed impaired renal function (60).Chelation therapy with intravenous Ca-EDTA was able to lower the body store of lead as indicated by a drop in urinary lead output (61). The rise in the circulatory inflammatory cytokines interleukin-1β, interleukin-6 and tumor necrosis factor-α and increase in hepatic stiffness and steatosis in battery factory workers after chronic exposure to lead were reduced following chelation therapy with intravenous CaNa2EDTA (62).

Earlier studies indicated that the liver is a major organ damaged, which has been corroborated by results of serum biochemistry. Adopting the Tandem Mass Tag™(TMT) approach, we noted that 41 out of 2,542 proteins were significantly altered correlated with the detoxification of lead and immune regulation by APL intervention. Through the analyses of DEPs GO function enrichment and KEGG enrichment, we observed that MHC Class II antigen, tetraspanin and RT1 class I antigen were up-regulated in expression in the APL-H group compared with the MOD group, which influenced the biological process of positive regulation of CD4-positive cells, alpha-beta T cell activation, and positive regulation of T cell mediated cytotoxicity. APL can improve MHC Class II antigen expression, positively regulate CD4+T cells by antigen-specific recognition of MHC molecule complex, activate T cell to execute effector functions, such as cytotoxicity, and provision of help to B cells and cytokine production response (63). Peptide-loaded MHC class II molecules, expressed on the surface of professional antigen-presenting cells, including dendritic cells, B cells, macrophages and thymic epithelial cells, are presented to antigen-specific CD4(+) T cells (64). RT1 class I antigen plays a role in the presentation of foreign antigens to the immune system. The data analysis indicates that APL can undermine lead-induced hepatorenal injury by positive regulation of immune processing and presentation similar to eradication of virus-infected cytotoxicity. Myeloperoxidase and glutathione S-transferase were found to be up-regulated in expression in the APL-H treated group. The heme protein myeloperoxidase is a key component of neutrophils found in the lysosomal azurophilic granules, and plays a central role in the innate immune response. The enzyme is released when lysosomes fuse with phagosomes after neutrophil activation, and facilitates, in neutrophils, the generation of hypochlorous acid which destroy microbes. Neutrophils lacking in myeloperoxidase exhibit a slower microbicidal action (65–67). Tetraspanins, transmembrane proteins on B cells, regulate antibody formation. Tetraspanins CD37 and CD81 are important for IgG formation whereas tetraspanin CD37 suppresses IgA formation. Tetraspanins CD9 and CD151 are important for humoral immune responses (68, 69). Xenobiotic metabolism/biotrans formation consists of 3 phases: phase I (modification), II (conjugation), and III (excretion). Glutathione S-transferase plays a crucial role as enzymes in phase II reactions in which electrophilic intermediates are conjugated with the antioxidant tripeptide glutathione to readily excretable forms. Glutathione S-transferase is protective against oxidative stress caused by formation of reactive oxygen/nitrogen species which produce molecular damage. It detoxifies anesthetic agents and its plasma and urinary activities are useful for monitoring toxicity to the liver and kidneys in patients subjected to general anesthesia and can serve as a prognostic biomarker (70, 71). Glutathione S-transferase reinstated in response to treatment with APL following decline due to lead exposure can thus elevate cytochrome P450 involved drug metabolism and metabolism of xenobiotics, accelerate oxidation and phosphorylation process, which influence the process of cellular oxidant detoxification, cellular response to toxic substance and cellular detoxification. The oxidative stress resulting from lead exposure can be mitigated by antioxidants (72). It is known that the water extract of *A. polytricha* exhibits a strong antioxidant actiivity (73). Nevertheless, the lead-detoxifying action of APL, as evidenced by the data on inhibition of lead accumulation and enhancement of glutathione S-transferases, is not linked to antioxidant activity because the bulk of antioxidative proteins are not upregulated. Selenized yeast, plant extracts, *Etlingera elatior* extract, ginger extract, flaxseed oil, myrrh emulsion, myrrh and turmeric, erdosteine and honey have all been demonstrated to elevate the activity of glutathione-S-transferase which has been downregulated due to lead exposure (74–82). Our findings on the upregulation of the detoxifying enzyme glutathione-S-transferase are in keeping with these previous observations. For MF analysis, the identified DEPs suggest that peroxidase activity, antigen binding, glutathione S-transferase activity, T cell receptor binding, MHC class II protein complex binding and threonine-type endopeptidase activity were activated in the APL-H group, which were consistent with BP analysis. The analysis implied that APL would activate the immune system to reinforce the cell protection mechanism to confront lead-induced toxicity. It is known that heavy metals other than lead, such as cadmium and mercury, also exert immunotoxicity (83–85). *A. polytricha* fruiting bodies were used for removing Hg and Cd (25, 86). Hence it is very likely that the immunoregulatory action of APL displayed in the present study is instrumental to the alleviation of heavy metal (Cd, Hg, and Pb) intoxication. Boatyard workers after chronic exposure to lead exhibited a nearly 9-fold higher lead level, reduced phagocytic activity, elevated interleukin-4, reduced interferon-γ, dysregulated subpopulations of Treg and Tc cells, and decreased cell-mediated immunity, increased death rate due to brain and lung cancers, indicating the profound effect of lead poisoning on immunity (45). *Moringa oleifera* leaf extract alleviated the immunotoxicity of lead by lowering the plasma levels of interleukin-2 and interferon-γ (87). In tilapia dietary chitosan overcame the immunotoxicity of lead as witnessed in enhanced phagocytic ability and lysozyme activity (88). Thus APL exerts a similar immune-related mechanism involving upregulation of a diversity of immunoregulatory proteins including myeloperoxidase, MHC Class II antigen, tetraspanin and RT1 class I antigen in its lead-detoxifying action. Genetic variations reflected in glutathione S-transferase gene polymorphism among different individuals is a determinant of susceptibility to lead toxicity indicating the paramount detoxifying role of glutathione S-transferase (89). The augmenting effect of APL on glutathione S-transferase facilitates the action of APL which also includes a biosorptive phemomenon.

To recapitulate, the present study evinces that APL plays a vital role in eliminating lead from the circulation. APL could mitigate weight loss engendered by lead exposure and suppress the levels of lead residue in blood and the liver and kidneys. Our findings indicate that APL attenuated liver damage after lead exposure by regulating the expression of proteins involved in detoxification and immune regulation. Based on the effects of APL, consideration can be given to APL as a therapeutic intervention for application in lead poisoning. *A. polytricha* also exhibits an array of health promoting activities encompassing hepatoprotective, renoprotective, neuroprotective, antioxidant, anticancer, antihyperlipidemic, antihypercholesterolemic, anti-inflammatory, anti-epileptic, and anti-HIV protease activities (18, 90–102). Hopefully future research will unravel more of its benefits to health.

## Data availability statement

The data presented in the study are deposited in the ProteomeXchange Consortium *via* the PRIDE partner repository, accession number PXD040493.

## Ethics statement

The animal study was reviewed and approved by Animal Ethical and Welfare Committee.

## Author contributions

SZ and TN conceived and prepared the manuscript. SZ, YG, and HW participated in glycoprotein purification. PW, WZ, DW, and XZ were responsible for animal experiment. YF analyzed the proteomics data. JW participated the manuscript editing. All the authors have read and agreed to the published vision of manuscript.
