# Supplementary material for: A novel mushroom (Auricularia polytricha) glycoprotein protects against lead-induced hepatoxicity, promotes lead adsorption, inhibits organ accumulation of lead, upregulates detoxifying proteins, and enhances immunoregulation in rats
Source: Front Nutr. 2023 Apr 6;10:1144346. doi: 10.3389/fnut.2023.1144346 (PMC10116064; doi:10.3389/fnut.2023.1144346)
Supplement: Supplementary file 1 [file Table_1.DOCX]

Table S1. Characteristics of APL in comparison with other mushroom glycoproteins

|  | Source | Molecular weight(Da) | N- terminal sequence | Inner amino acid sequences | Monosaccharide composition |
| --- | --- | --- | --- | --- | --- |
| APL | *Auricularia polytricha* | Mw=2.52×10^5^ | HDDMGMSAMM | LLDQGQAGDNVGLLLR,  HYAHVDCPGHADYVK,  AYDQIDAAPEEK,  GYRPQFYFR,  TVGAGVVAK | Man: Rha: Glu: Gal: Xyl: glucuronic acid: galacturonic acid =27.8:8:19.3:22.7:8.7:30:9 |
| APPI | *A. polytricha* | Mw= 9.213×10^5^  Mn=5.568×10^5^  Mp= 1.057×10^6^ | DLYEVVEGEI | VQNVGNGVLLGFHGR  HQTSGDQVTSSTQHSFR | Ara: Gal: Glc: Xyl: Man =1:4.4:15.4:38.3:46.2 |
| APPII | *A. polytricha* | Mw= 6.340×10^5^  Mn= 7.693×10^4^  Mp= 9.547×10^5^ | VPSSMVVVVG | GTPSSYIDNLTFPK  ELATGQNGFGYAGSSFHR | Ara: Gal: Glc: Xyl: Man =1:71.5:99.2:10:5.1 |
| PSI | *Pleurotus citrinopileatus* | Mw= 1.216 × 10^6^ | DLEQVVEGDW | IQDKEGIPPDQQR,  ISGLIYEETR,  KNGEILGGSWMVGAK | Ara: Man: Glu: Gla =1:6.2:6.3:67.2 |
| PSII | *P．citrinopileatus* | Mw =1.608 × 10^4^ | KLSEGWERPP | SSEREDLWQSTHVGHDEFSK,  DGSLTGTYHSNV  GEVPPTYHLSGR,  EDLWQSTHVGHDEFSK | Xyl: Glu: Gal=1:83.9:4.2 |
| GFPP | *Grifola frondosa* | Mw =9.6 × 10^4^ | APPGMHQKQQ | LVSLSCDPNHTFSIDGHSLTVIEADSVNLKPHTVDSIQIFAAQR,  SLYDVDDDSTVITLADWYHLAAR,  QAILVNDVFPSPLITGNKGDR, VGPAIPTADATLINGLGR,  YSFVLNADQDVDNYWIR,  SINTLNADLAVITVTK,  NFDGGVNSAILR |  |
