# Supplementary material for: A novel mushroom (Auricularia polytricha) glycoprotein protects against lead-induced hepatoxicity, promotes lead adsorption, inhibits organ accumulation of lead, upregulates detoxifying proteins, and enhances immunoregulation in rats
Source: Front Nutr. 2023 Apr 6;10:1144346. doi: 10.3389/fnut.2023.1144346 (PMC10116064; doi:10.3389/fnut.2023.1144346)
Supplement: Supplementary file 3 [file Image_1.PDF]

Supplementary Material

Lead eliminating activity of Auricularia polytricha glycoprotein protects against lead acetate-induced hepatorenal toxicity in rats via positive immunoregulation pathway: the first demonstration

Shuang Zhao[1*], Yi Gao[2], Hexiang Wang[3], Yangyang Fan[1], Pan Wang[1], Wenting Zhao[1], Jack Ho Wong[4], Dan Wang[1*], Xiaoyan Zhao[1*], Tzi Bun Ng[5*]

*** Correspondence:** Corresponding Author: wangdanjgs@163.com (D Wang), xiaoyangzhao001@163.com (X Zhao), tzibunng@cuhk.edu.hk (TB Ng)

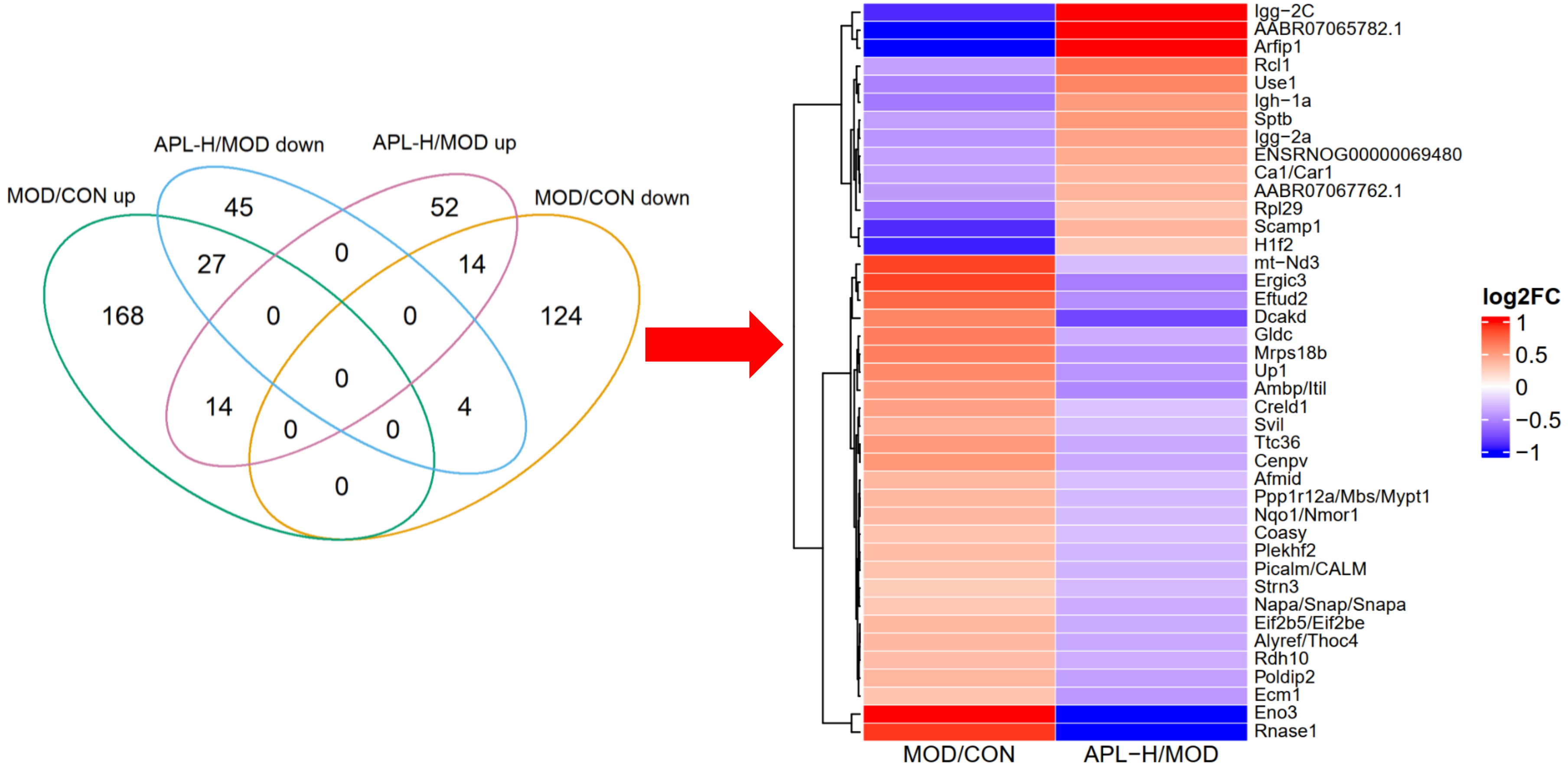

Supplementary Figure 1. Normalized DEPs analysis of APL-H treatment.
